# Supplementary material for: Effects of Livestock Grazing on Spatio-Temporal Patterns and Behaviour of Reeves’s Pheasant Syrmaticus reevesii
Source: Animals (Basel). 2022 Oct 28;12(21):2968. doi: 10.3390/ani12212968 (PMC9656763; doi:10.3390/ani12212968)
Supplement: Supplementary file 1 [file animals-12-02968-s001.zip › Supplementary Materials.pdf]

Supplementary Materials

Table S1: Specifications of the study design in each study area, including the number and duration of camera traps.

Study design	Study area	
	LKS	ZHS
Camera trap model	Ltl Acorn 6310 WMC, Zhuhai, China	Ltl Acorn 6310 WMC, Zhuhai, China
Sensitivity of the sensor	Medium	Medium
Pictures per photo series	3 pictures and 1 video	3 pictures and 1 video
Delay between photo series	30s	30s
Camera trap installation height	0.3-0.4 m	0.3-0.4 m
Field measurements to allow estimation of elevation and vegetation	Yes	Yes
Length of deployment periods	12 months	12 months
Number of camera traps	35	37
Number of camera traps in normal operation	27	33
Number of damaged camera traps	1	1
Number of camera traps stolen	7	3

Table S2: Covariate collinearity analysis based on variance inflation factor

Variable	VIF	df	GVIF^(1/(2*Df))
Distance to the nearest impervious road	4.5	1	1.32
Distance to the nearest settlement	4.88	1	2.21
Distance to the nearest cultivated land	1.33	1	1.15
Distance to the nearest water	3.37	1	1.84
Elevation	1.87	1	1.37
Livestock encounter rates	1.73	1	1.32
People encounter rates	1.15	1	1.1

Table S3: Behavioural ethogram used in behavioral analysis of Reeves's Pheasant

Behaviour	Definition
Foraging	Head down cropping the seed or jump up to eat grass.
Vigilance	Head up on alert posture, even between feeding bouts.
Locomotion	Running or fast walking without feeding.
Comfort behavior	Standing or sitting with the neck folded and head on shoulder, simply laying down, or preening (<5%).
Others	Less common behaviors, sparring, brood, provisioning, courtship (<5%).

Table S4: A two-tailed unpaired two-sample Wilcoxon test was used to examine the difference in camera trap average sampling efforts of ZHS and LKS during breeding season, non-breeding season and the whole year.

Stage	Camera trap days				w	*p*
	LKS		ZHS			
	mean	se	mean	se		
Breeding season	114.16	46.71	116.83	49.52	514.00	0.56
Nonbreeding season	170.71	50.60	179.31	42.49	402.50	0.47
A whole year	259.97	90.53	268.76	90.79	558.00	0.68

Table S5: Results of fitting single-species occupancy models to the detection histories of Reeves's Pheasant. We report the best-supported models (ΔAIC < 2) in both breeding and nonbreeding season. The set of competing models for each species consisted of 64 models. (DNR = distance to the nearest road, DNS = distance to the nearest settlement, Ele = elevation, LER = livestock encounter rates).

Life history	model names	K	AIC	ΔAIC	AICwt
nonbreeding season	psi(DNR)p(location+vegetation)	5	1446.48	0.00	0.15
	psi()p(location+vegetation)	4	1447.02	0.54	0.11
	psi(DNR+DNS)p(location+vegetation)	6	1447.55	1.07	0.09
	psi(LER+DNS)p(location+vegetation)	6	1447.57	1.09	0.08
	psi(DNR)p(location+Ele)	5	1448.39	1.92	0.06
breeding season	psi()p(DNR+DNS)	4	1813.62	0.00	0.39
	psi(location) p(DNR+DNS)	5	1815.08	1.47	0.19
	psi(LER) p(DNR+DNS)	5	1815.27	1.66	0.17

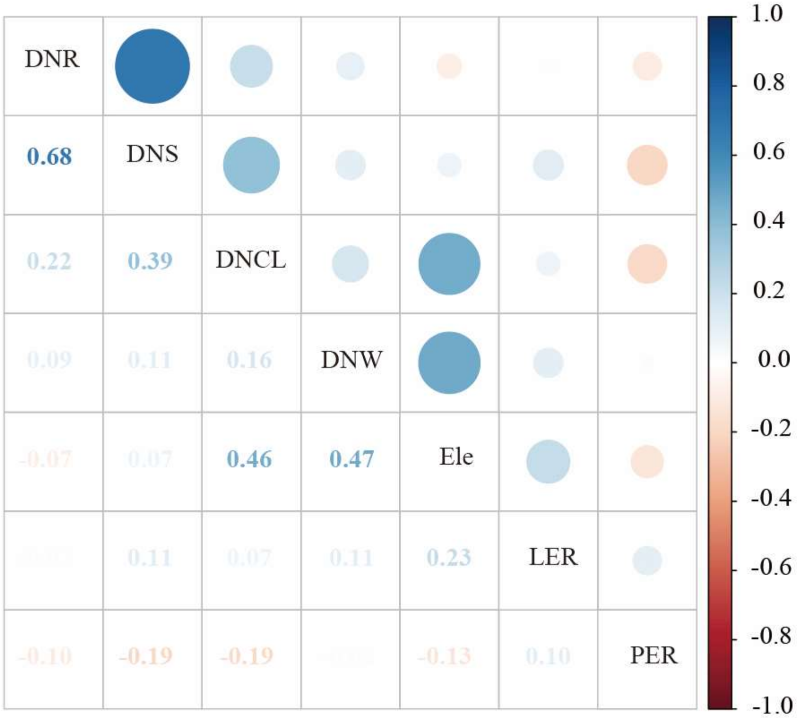

Figure S1: Correlation coefficients between the different forest disturbance variables. Numbers on the left reflect pairwise correlation coefficients; circle size and transparency denote the magnitude of correlation. The letters on the diagonal indicate the abbreviation of the covariates (DNR = distance to the nearest road, DNS = distance to the nearest settlement, DNCL= distance to the nearest cultivated land, DNW =distance to the nearest water, Ele = elevation, LER = livestock encounter rates, PER = People encounter rates).

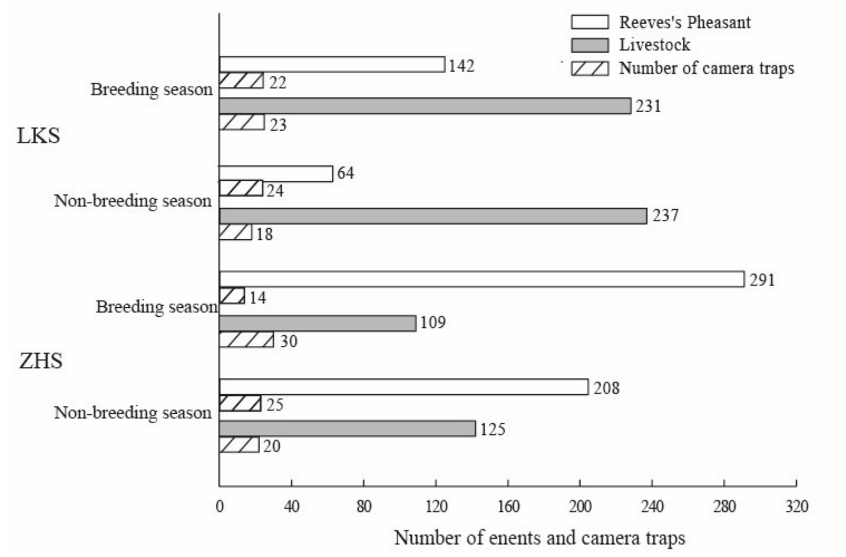

Figure S2: The total number of camera traps (CTs) and total number of events using camera traps in LKS and ZHS, China, from July 2018 to July 2019.

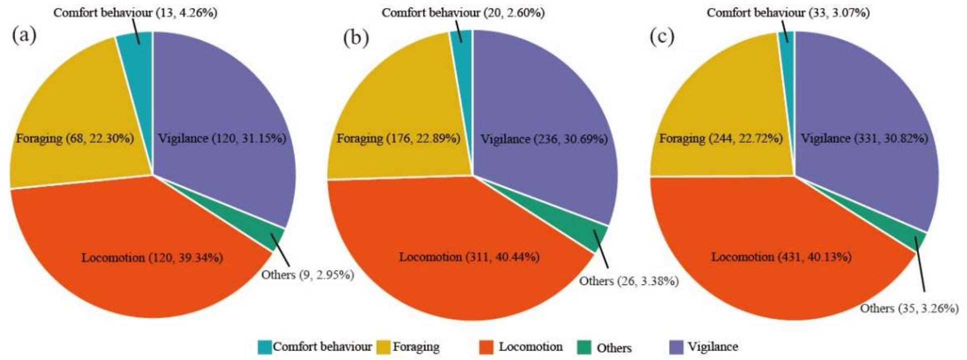

Figure S3: Occurrence of the behavioral categories in LKS, ZHS, and both. The five parts of the pie chart represent the five behavior categories, and the numbers in parentheses indicate the number and percentage of behavior occurrences, respectively. (a) LKS, (b) ZHS, (c) both.
